# Clinical Applications of the Intercostal Artery Perforator Flap for Trunk Reconstruction

**DOI:** 10.1055/a-2058-7927

**Published:** 2023-05-29

**Authors:** Young Jun Kim, Woo Young Choi, Ji Seon Cheon, Min Hyub Choi

**Affiliations:** 1 Department of Plastic Reconstructive Surgery, Chosun University College of Medicine, Gwang-ju, South Korea

**Keywords:** donor site, intercostal artery perforator flap, reconstruction, trunk defects

## Abstract

**Background** Trunk defects can occur because of surgical site infections after spinal surgery, resection of malignant tumors, or trauma. Herein, we present our experience of using intercostal artery perforator (ICAP) flaps to reconstruct trunk defects without noteworthy complications. Fourteen patients underwent reconstruction with ICAP flaps between March 2015 and March 2019.

**Methods** Patients' data, including age, sex, the cause of the defect, defect size, perforator location, flap size, complications, and follow-up period, were retrospectively reviewed. The mean age of the patients was 56.5 years (range, 19–80 years). All operations were performed after the results of bacterial culture from the wound showed no microbial growth. We found reliable perforators around the defect using Doppler ultrasonography. The perforator flaps were elevated with a pulsatile perforator and rotated in a propeller fashion to the defects. We performed five dorsal and two lateral ICAP flaps. The mean flap dimensions were 12 × 5.5 cm ^2^ (range, 6 × 5 to 18 × 8 cm ^2^ ).

**Results** Primary closure of the donor site was performed. Marginal congestion was observed as a complication in one case, but it healed with no need for revision. The mean follow-up period was 8 months. All patients were satisfied with the surgical outcomes.

**Conclusion** ICAP flaps can be easily mobilized, thereby reducing donor site morbidity without sacrificing the underlying muscles for trunk reconstruction. Therefore, these flaps are useful options for the reconstruction of trunk defects.

## Introduction

The reconstruction of complicated trunk defects involving bone or spinal prosthesis exposure is challenging. Trunk defects can occur due to surgical site infections after spinal surgery, resection of malignant tumors, or trauma. Trunk defects are generally repaired using traditional muscle or musculocutaneous flaps, skin grafts, and free flaps. 1 Skin grafting is a simple and easy method; however, it has limitations when used for complicated wounds. Traditional muscle or musculocutaneous flaps require the sacrifice of donor muscles. In large defects, a free flap can be used. However, performing flap elevation in a patient in the prone position is technically difficult, and identifying an appropriate recipient vessel is also challenging. 2

As numerous studies have assessed perforator flaps, which have been widely used clinically, trunk defects are currently repaired using various skin flaps based on a diverse range of perforators. A sufficiently large flap can be prepared with a single perforator, and the flap can be rotated in a propeller fashion. Thus, designing a flap is easier if a perforator can be located around a defect, because the presence of a nearby perforator makes defect coverage more straightforward. Conventional flaps using intercostal artery vessels that form an arcade between the aorta and the internal mammary vessels with numerous perforators have been used. 3 Furthermore, Hamdi et al conducted clinical and anatomical studies on intercostal artery perforator (ICAP) flaps in the 2000s. 4 5 This study aimed to document the clinical usefulness of ICAP flaps for complicated truncal defects.

## Methods

Fourteen patients underwent reconstruction with ICAP flaps between March 2015 and March 2019. Data, including age, sex, the cause of the defect, defect size, perforator location, flap size, complications, and follow-up periods, were retrospectively reviewed ( Table 1 ). The mean age of patients was 56.5 years (range, 19–80 years). The mean follow-up period was 8 months. The etiologies of the defects were postoperative infection or dehiscence in 12 patients, gunshot injury in 1 patient, and skin infection caused by *Vibrio* in 1 patient. All reconstructions were performed after the bacterial culture of the wound was negative.

**Table 1:** Patient data

No.	Age (y)	Sex	Cause of defect	Defect size (cm ^2^ )	Flap size (cm ^2^ )	Follow-up (mo)	Complications	Perforator
1	66	M	Postoperative dehiscence	6 × 5	15 × 5.5	10	None	DICAP
2	67	M	Postoperative dehiscence	8 × 4	11 × 4.5	14	None	DICAP
3	65	M	Postoperative dehiscence	12 × 4	15 × 5	9	None	DICAP
4	68	M	Postoperative dehiscence	6 × 3	10 × 4	10	None	DICAP
5	19	M	Postoperative dehiscence	5 × 3	6 × 5	8	None	DICAP
6	32	M	Gun shot	19 × 8	18 × 8	11	None	LICAP
7	45	M	Bacterial infection	8 × 6	10 × 7	8	Marginal necrosis	LICAP
8	69	F	Postoperative dehiscence	1 × 2	3 × 4	6	Spinal abscess	DICAP
9	77	M	Postoperative dehiscence	8 × 3.5	16 × 4	4	None	DICAP
10	80	M	Postoperative dehiscence	3 × 4	12 × 4.5	6	None	DICAP
11	49	M	Postoperative dehiscence	5 × 6	6 × 4	8	None	DICAP
12	66	F	Postoperative dehiscence	6 × 4		6	None	DICAP
13	20	M	Postoperative dehiscence	3 × 2	4 × 3	9	None	DICAP
14	69	M	Decortication of lung	10 × 3	13 × 5	3	None	LICAP

Abbreviations: DICAP, dorsal intercostal artery perforator; F, female; LICAP, lateral intercostal artery perforator; M, male.

The mean defect size was 49 cm ^2^ (range, 15–152 cm ^2^ ). Patients with small defects that could be managed using primary closure or skin grafting were excluded. The study was approved by the Institutional Review Board of the Chosun University Hospital (approval number: 2020-06-009), and written informed consent was obtained from all patients for participation and publication of their data.

### Surgical Technique

In all patients, we found perforators adjacent to the defect using a hand-held Doppler ultrasound device before flap design and skin incision. We attempted to locate the perforator as close to the defect as possible. The flap was designed in an elliptical pattern.

The long axis of the flap was designed such that the distance between the perforator and the distal portion of the flap was longer than that between the perforator and the furthest part of the defect, in order to facilitate tensionless closure ( Fig. 1 ).

**Fig:**
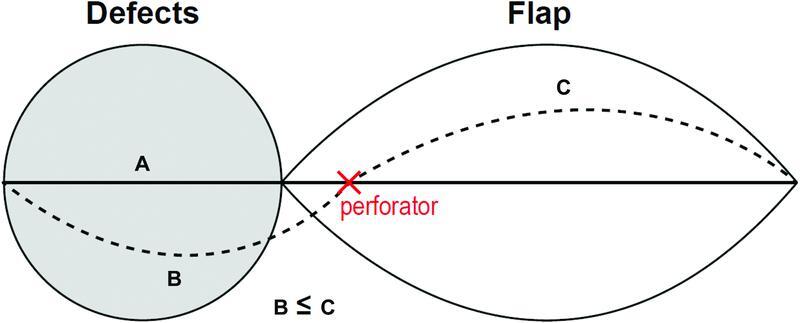
**1** Flap design. The flap was designed with an elliptical shape. ( **A** ) Long axis of the defect. ( **B** ) Distance between the perforator and the distal portion of the flap. ( **C** ) Distance between the perforator and the furthest part of the defect.

We used dorsal ICAP (DICAP) flaps to reconstruct midline back defects and lateral ICAP (LICAP) flaps to reconstruct the lateral chest wall based on the location of the defect. After a skin incision was made, we continued the dissection down to the muscle fascia. The flap was elevated in a subfascial manner. One perforator with visible pulsation was selected as the pedicle perforator, and the other perforators were ligated. The pedicle was dissected sufficiently deep to obtain a sufficient rotation arc, and the flap was transferred in a propeller fashion. Proper circulation in the flap was verified by capillary refill and a Doppler examination after flap rotation. After the flap was inset, drains were placed beneath the flap. The donor sites were primarily closed in all patients.

## Results

We performed reconstructions using 11 DICAP flaps and 3 LICAP flaps. The mean flap dimensions were 12 cm × 5.5 cm, and the mean follow-up period was 10 months. In a patient with skin infection caused by *Vibrio* , congestion was noted in the distal flap margin, and the flap was allowed to heal by secondary intention. However, marginal necrosis occurred because of excessive tension due to severe scarring caused by *Vibrio* infection around the defect. One late infection was noted because of the placement of spine fixation devices 4 months after flap reconstruction. A skin incision was made along one side of the flap margin, and a neurosurgeon changed the spine fixation devices. No issues were noted regarding flap survival. All donor sites were closed, and no complications developed. None of the patients developed complications during motion at the last follow-up visit.

### Case 1

A 66-year-old man with a 10-year history of hypertension visited our department because of a soft tissue defect in the posterior part of his neck. At another hospital, he had undergone posterior cervical spine fusion surgery to treat spinal stenosis, which was diagnosed 5 months previously. However, the surgical site became infected, and he underwent bilateral V-Y flap coverage surgery to cover the defect at the previous hospital. Ten days postoperatively, the flap became necrotic, and a 6 cm × 5 cm defect was noted in the midline of the cervical area. The muscles were exposed to a significant amount of discharge, which was suspected to be the result of an infection ( Fig. 2A ). Intravenous Maxipime (cefepime) was administered based on the results of a wound culture that identified *Pseudomonas aeruginosa* , debridement of the unhealthy tissue was performed, and vacuum-assisted dressing was applied. After controlling the infection at the wound site, reconstruction using a DICAP flap was performed. The flap, measuring 15 cm × 5.5 cm, was rotated in a propeller fashion to cover the defect. The donor site was then closed primarily ( Fig. 2B ). No complications associated with the flap or donor site were observed. At the 3-month follow-up visit, the patient had recovered without any complications ( Fig. 2C ).

**Fig:**
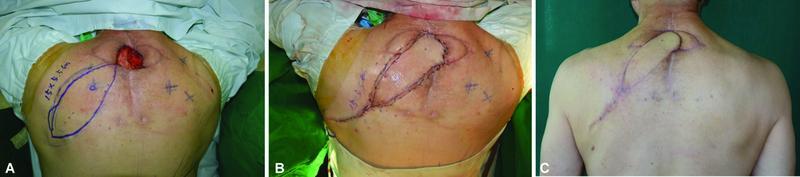
**2** Case 1. ( **A** ) A 6 cm × 5 cm defect due to surgical site dehiscence after bilateral V-Y advancement flap coverage in a 66-year-old man. A DICAP flap was done in a propeller flap fashion. ( **B** ) Immediately after surgery, the flap appeared pinkish in color. Doppler findings were normal. ( **C** ). A healthy wound was noted at a 3-month outpatient follow-up.

### Case 3

**Fig. 3:**
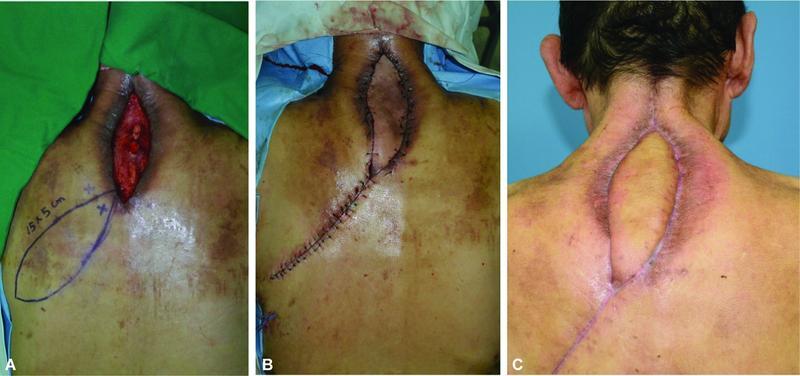
Case 3. ( **A** ) A 15 cm × 5 cm defect after surgical debridement. ( **B** ) Flap inset with DICAP propeller flap coverage. ( **C** ) A healthy-looking wound 4 months after surgery.

A 65-year-old man with a 5-year history of diabetes mellitus underwent posterior cervical spine fusion surgery in the neurosurgery department because of a traumatic cervical fracture. After 1 month, he was transferred to our department because of a surgical site infection and a soft tissue defect on his posterior neck. The defect size was approximately 14 cm × 4 cm, and had undermined edges with exposure of the spinous process and accompanying peripheral infection. After treatment with appropriate antibiotic therapy and vacuum-assisted closure, reconstruction using a DICAP flap was performed. The defect was covered with an elliptical rotational flap that measured 15 cm × 5 mm. Primary closure of the donor site was performed. No complications associated with the flap or donor site were noted ( Fig. 3 ).

### Case 6

**Fig. 4:**
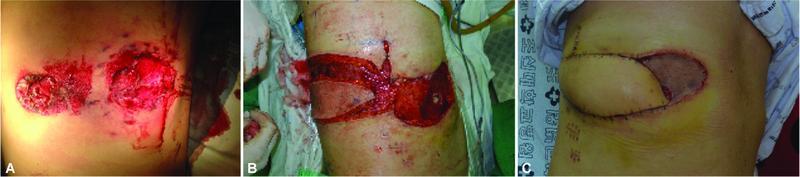
Case 6. ( **A** ) Soft tissue defects on the right lateral chest in a 32-year-old man after the removal of bullets from a gunshot injury (shotgun). ( **B** ) A LICAP flap on the same side was performed with a split-thickness skin graft on the remaining defect. ( **C** ) Postoperative image.

A 32-year-old man was admitted to the emergency department of our hospital with complaints of bleeding and a soft tissue defect on the right lateral part of his chest due to a gunshot. Although debridement and removal of the bullet were performed by a general surgeon, the wound became worse with marginal redness and discharge of pus, which were suggestive of an infection. The patient was transferred to our department for infection control and reconstruction. The defect size was 19 cm × 8 cm in the right lateral chest area, and the seventh rib was exposed ( Fig. 4 ) Debridement, antibiotic therapy, and wound care were initiated. After controlling the infection, one-stage reconstruction using a LICAP flap on the same side was performed. An elliptical flap, measuring 18 cm × 8 cm, was designed by pinching the skin over the donor site to determine whether the donor site could be primarily closed. The flap was rotated in a propeller fashion to repair the defect, and the shallow wound area was covered with a skin graft for a flap that was not designed to invade the midline. The donor site was closed primarily. The flap survived without any complications, and the skin graft completely healed.

### Case 13

**Fig. 5:**
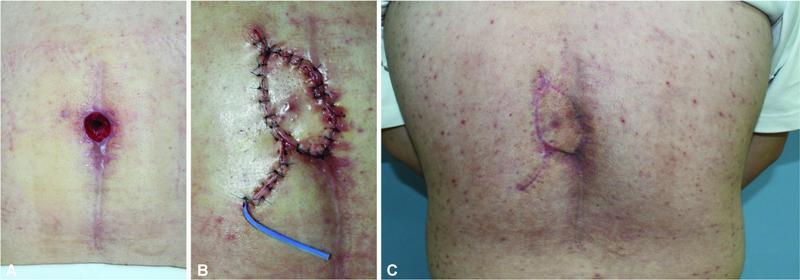
Case 13. ( **A** ) Wound dehiscence after posterior thoracic spine fusion in a 20-year-old man. ( **B** ) DICAP flap coverage. ( **C** ) A healthy wound at a 6-month outpatient follow-up.

A 20-year old man with no underlying diseases underwent posterior thoracic spine fusion surgery in the neurosurgery department because of a traumatic thoracic spine fracture. After 3 months, he was transferred to our department because of wound dehiscence resulting from an infection of the fixation implant. The defect was small, approximately 3 cm × 2 cm, but it had an undermined edges with exposure to the spinous process and fixation materials. After wound debridement, irrigation with appropriate antibiotic therapy, and potadine gauze packing dressing, reconstruction using DICAP flap was performed. The defect was covered with an elliptical rotational flap that measured 4 cm × 3 cm. Primary closure of the donor site was performed. No complications associated with the flap or donor site were noted ( Fig. 5 ).

## Discussion

The reconstruction of complex soft tissue defects in the trunk is challenging. Conventional muscle or musculocutaneous flaps, such as the latissimus dorsi, trapezius, and paraspinous muscle flaps, are useful options for trunk reconstruction. 1 However, donor site complications, including hematoma and seroma, may occur because of the need for excessive dissection, long surgery times, and functional loss caused by muscle sacrifice. 1 A free flap is a good option for covering large defects. However, it is not technically easy to perform flap elevation with the patient in the prone position. 2 Free flaps are difficult to perform in patients with compromised vessels and severe comorbidities. They also require a long surgical time and lengthy postoperative hospitalization.

With the development of perforator flaps and concept of the perforasome, many previously described musculocutaneous flaps could be harvested as perforator flaps with preservation of the underlying muscles. 4 5 6 Perforator flap surgery does not require excessive dissection or muscle sacrifice; therefore, the surgery is shorter, and the complications associated with excessive dissections are avoided. A less invasive approach is advantageous for treating older patients and patients with comorbidities. Furthermore, because the major muscles in the trunk are conserved, functional loss in the donor muscles is avoided.

The intercostal artery has long been used for skin flaps that are not perforator flaps. In 1974, Dibbell reported that an intercostal flap including the anterior cutaneous nerve was used to provide sensation in cases of sacral reconstruction. 7 Anatomical studies by Kerrigan and Daniel led to a better understanding of the clinical indications and surgical technique. 3 However, these intercostal neurovascular flaps have some disadvantages. First, pedicle dissection is technically difficult, and there is a high risk of iatrogenic pneumothorax during intercostal space dissection. Second, there is a risk of the pedicle being susceptible to compression. 8 Therefore, by using a flap based on an ICAP to reconstruct a trunk defect, we can overcome these risks and can benefit from the advantages of a perforator flap.

**Fig. 6:**
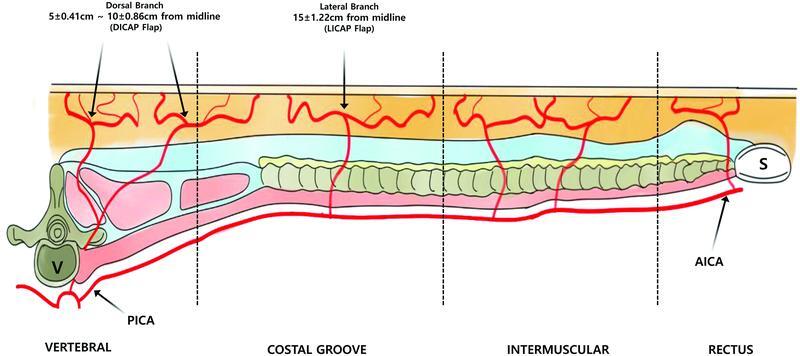
Schematic illustration of posterior intercostal artery (PICA), DICAP, and LICAP, representing four segments of the intercostal space: vertebral, costal groove, intermuscular, and rectus. The branches of the PICA that supply DICAP and LICAP flaps are shown. A, aorta; AICA, anterior intercostal artery; DICAP, dorsal intercostal artery perforator; LICAP, lateral intercostal artery perforator; S, sternum; V, vertebra.

The intercostal vessels form an arcade between the aorta and the internal mammary vessels, with numerous perforators. The arteries are classified into four segments, namely, the vertebral, intercostal, intermuscular, and rectus. 4 5 ICAP flaps are named according to their site of origin. A flap with a perforator originating in the intervertebral segment is called a DICAP flap, one with a perforator originating in the intercostal segment is called a LICAP flap, and a flap with a perforator originating in the intermuscular or rectus segment is known as an anterior ICAP (AICAP) flap 3 ( Fig. 6 ). DICAPs supplying the skin of the back usually arise at a mean distance of 5 ± 0.41 cm and 10 ± 0.86 cm from the midline. LICAPs arise at a mean distance of 15 ± 1.22 cm from the midline. 9

Perforators were abundantly present in the body; thus, they could be easily found around the defect. Based on the concept of the freestyle flap, ICAP flaps can be harvested to cover truncal defects that extend from the lower neck to the lower abdomen and the lumbosacral area. 10 Since Minabe and Harii, and Hamdi et al conducted cadaver dissection studies and angiographic studies to map DICAPs and LICAPs, many studies have reported the use of ICAP flaps to manage defects caused by cancers, pressure sores, or infected wounds. 4 5 11 We used a perforator that extended from the posterior intercostal artery at the 3rd to 11th position and originated from the aorta to reconstruct back midline defects.

LICAP flaps can measure up to 25 cm × 20 cm in size; however, since the maximum width capable of primary closure is 12 cm, caution must be exercised during flap design. In this study, the flap was designed as an 8-cm-wide LICAP flap to reconstruct the lateral chest wall defect. A flap of sufficient size can be obtained for the primary closure of the donor site. The AICAP is located 1 to 3 cm lateral to the sternal border and is used for the reconstruction of sternal, breast, and thoracic defects. In 2006, Hamdi et al reported the use of V-Y advanced-type AICAP flaps to repair anterior chest defects caused by tumor excision. 5

The LICAP is located at the junction of the midaxillary line and the lower border of the corresponding rib in the 3 ^rd^ to 11 ^th^ intercostal space. Because of the short pedicle length, it is possible that some breast defect reconstructions might require only the lateral quadrant. 4 The DICAP is placed up to 5 cm lateral to the midline posteriorly. Therefore, DICAP flaps were used to treat midline defects in the back. De Weerd and Weum reported using the DICAP to close cervicothoracic midline defects after spinal surgery. 12 Their study was similar to our study because ICAP flaps were used for the management of infected wounds, although it differed in the use of the medial branches of DICAP and a rotational-style flap design.

Our study included wounds caused by postoperative wound dehiscence and gunshot injuries. In six patients, foreign bodies, such as spinal prostheses or bullets, remained. Coverage with an ICAP flap on the trunk defect allowed padding of the exposed structures, obliteration of dead space, and tension-free closure of the wound.

The ICAP flap is an important option for reconstructing challenging trunk defects. Harvesting these flaps without sacrificing the underlying muscle reduces donor site morbidity and provides more freedom to compose and tailor the flap without the risk of pneumothorax. ICAP flaps have a high capacity for mobilization; therefore, as discussed earlier, this flap is useful for reconstructing trunk defects, such as midline defects of the back, lateral chest defects, and breast lateral quadrant defects. Furthermore, as in some of our cases, in defects with scar and chronic infections, suspected of weakening the vascular system—even if the defects are small and primary closure seems possible—a well-vascularized ICAP flap can be used as a zone of injury to help recover the ICAP.

Some limitations of this study were the small number of cases and the fact that AICAP flaps were not used in any patients. The follow-up period was also short; therefore, regular management of the patients' donor site morbidities was required. In addition, in patients with spine devices, such as in the case of late infection, the occurrence of infection should be monitored during long-term follow-up. Unlike the previously described use of intercostal neurovascular island skin flaps, we did not reconstruct the defects using sensate flaps. In the future, long-term follow-up will be needed to investigate the occurrence of postoperative infections and donor site morbidity. Further studies on designs that can be used to restore larger defects are also needed.

It is also difficult to evaluate whether a perforator is healthy or can ufficeently supply blood to the vessel territory if a flap is designed using only a hand-held Doppler device. Furthermore, Doppler ultrasound is easy to use but is operator-dependent and has shown low sensitivity for the identification of perforators and high interuser variation.

Currently, noninvasive computed tomography angiography is used primarily to evaluate vascular status. It also provides better information regarding atherosclerotic changes in vessels, 13 and in flap planning and harvesting, infrared thermal scanning serves as a noninvasive method of assessing perforator location and quality and thus assists in surgical decision-making. 14 In a later study, the flap survival rate should be improved using a tool to validate the vascular status.

Moreover, a meta-analysis comparing the actual consistency or survival rate between the perforators detected with a hand-held Doppler device and a thermal scanner or computed tomography angiography would contribute to the field by enhancing our knowledge regarding noninvasive approaches to patients in all stages (preoperative, surgical, and postoperative), which is currently a topic of active research.

ICAPs have an anatomically consistent structure from the 4 ^th^ to the 11 ^th^ intercostal space and are present in many parts of the body, especially around defects. ICAPs are also abundant in indirect and direct linking vessels that connect perforasomes. Therefore, when determining the size and shape of a flap, it is possible to easily find and design ICAP flaps around trunk defects. ICAP flaps can enable satisfactory restoration in patients with a wide variety of defects in terms of size, site, and the presence or absence of bone or spinal device exposure.
